# Annual Movement Patterns of Endangered Ivory Gulls: The Importance of Sea Ice

**DOI:** 10.1371/journal.pone.0115231

**Published:** 2014-12-31

**Authors:** Nora C. Spencer, H. Grant Gilchrist, Mark L. Mallory

**Affiliations:** 1 Department of Biology, Acadia University, Wolfville, Nova Scotia, Canada; 2 Environment Canada, National Wildlife Research Centre, Carleton University, Ottawa, Ontario, Canada; Institute of Ecology, Germany

## Abstract

The ivory gull (*Pagophila eburnea*) is an endangered seabird that spends its entire year in the Arctic environment. In the past three decades, threats from various sources have contributed to a >70% decline in Canada. To assess the annual habitat needs of this species, we attached satellite transmitters to 12 ivory gulls on Seymour Island, Nunavut in 2010, which provided up to four breeding seasons of tracking data. Analysis of migratory behaviour revealed considerable individual variation of post-breeding migratory route selection. Ivory gulls traveled a median of 74 days during post-breeding migration, but only 18 days during pre-breeding migration. In contrast to predictions, ivory gulls did not use the Greenland coast during migratory periods. Ivory gulls overwintered near the ice edge in Davis Strait, but also used the Labrador Sea in late February and March. We suggest that the timing of formation and recession and extent of sea ice plays a large role in ivory gull distribution and migratory timing.

## Introduction

The Arctic marine environment is changing in response to from a variety of anthropogenic activities [1]. Indeed, climate trends and models show global temperatures increasing with anticipated reductions in sea ice cover, which may be accelerating interest in resource exploitation in the Arctic [2], [3]. Warming air temperatures and thinning sea ice mean that annual ice is replacing marine habitats that traditionally are covered in multi-year ice [4], [5]. In fact, in 1987, 57% of sea ice in the Arctic basin was ≥5 years old; however in 2007 that number had dropped to 7% [6]. The Arctic is changing more rapidly in response to global warming than any other area of the world; therefore, continued assessment and monitoring of this sensitive environment and how warming trends are influencing Arctic flora and fauna is pertinent [5]. Species that may be particularly affected should be pagophilic organisms that rely on the annual integrity and extensive sea ice for various life functions [5], [7], [8]. Arctic seabirds have proven to be a particularly effective group of organisms with which to monitor variation in the Arctic environment, as they feed at different trophic levels in Arctic food chains and rely on sea ice habitats for foraging opportunities and consequently their reproductive success and habitat use reflects conditions of the food webs on which they depend [2], [9].

Of the Arctic seabird species that breed in Canada, the ivory gull (*Pagophila eburnea*) should be an excellent bioindicator of the effects of global warming because it has year-round affinities for ice habitats [10]–[13]. To date we know relatively little about the species' breeding biology or its habitat needs in Canada [14], although there have been recent large declines (∼70%) in the Canadian breeding population [15]. It is an endangered species listed under the Species at Risk Act (SARA) in Canada and listed as near-threatened by the International Union for Conservation of Nature (IUCN) red list [16]–[17]. Breeding populations are found in Svalbard (Norway), Greenland (Denmark) and Russia [18], as well as the Canadian high Arctic [15]. During the non-breeding season, Canadian ivory gulls are observed in Davis Strait and the Labrador Sea in winter [10], although our information on these locations is very limited. At this time, the ivory gull is most likely found over pack ice of 70–90% ice concentration, as well as along ice edges, but is rarely observed over open water >5 km from ice [14]. Little is known of the migratory behaviour, movements and key habitat needs of the ivory gull away from their breeding colonies [15]–[16], although new information is emerging on post-breeding movements of birds from Greenland, Svalbard and Russia [19]. Given that Arctic environments are changing and evidence already suggests deleterious effects on other pagophilic wildlife (e.g. polar bears, *Ursus maritimus*) [20], additional research is required to understand the annual habitat needs and behaviours of ivory gulls in relation to the marine environment, to better manage this endangered species and to identify possible actions to promote its recovery and protection [21]. However, given the remote location and limited accessibility of the ivory gull, direct observation of gulls through field research is logistically and financially challenging. Consequently, we used satellite telemetry to define year-round distribution, migratory movements and behaviour of the species (as reviewed in Burger and Shaffer [22]). Satellite telemetry is a widely used technique in seabird studies, providing information on bird behaviour at times when they cannot practically be observed from land or sea [14], [19], [22]–[25].

The breeding locations and suspected wintering locations for Canadian ivory gulls have been identified previously [10], [14], [26], [27]. However, information on migration timing and movements is largely speculative, although many Canadian birds migrate along the Greenland coast in spring and fall [27]. We anticipated that sea ice distribution and timing of formation were the primary factors influencing how ivory gulls move from their breeding colony in the central Canadian high Arctic to their suspected wintering area in Davis Strait and back to their breeding colony the following year. Based on this hypothesis, we tested three predictions with satellite data from ivory gulls: 1) gulls would use primarily marine habitats and travel routes during migration, not terrestrial routes; 2) gulls would migrate primarily over ice, and would not migrate in advance of its formation; and 3) wintering locations over ice would be close to the floe edge, because this area was more likely to provide foraging options (e.g., hooded seal *Crystophora cristata* whelping patches [10], [14]).

## Methods

### Ethics Statement

All research was conducted following animal care committee approval for Canadian Wildlife Service Banding Permit number 10694 as well as Canadian Wildlife Service Scientific Permit NUN-SCI-09-02 and Nunavut Wildlife Research License WL2010-032.

### Field Methods

**Figure 1:**
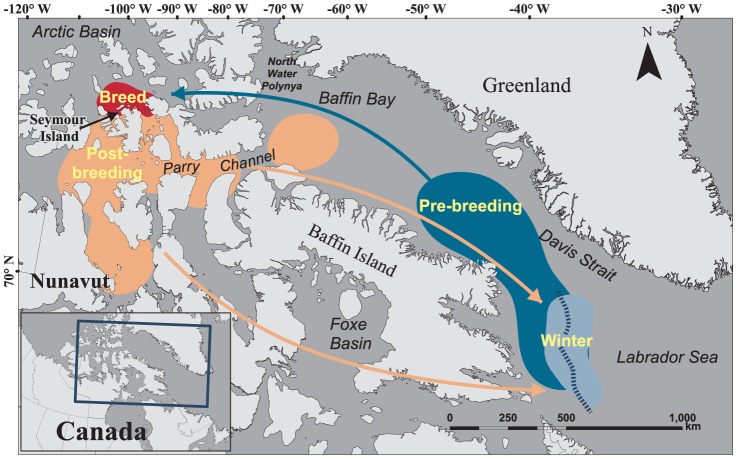
Annual distribution of the Canadian ivory gull. The 50% kernels represent distribution during breeding (red), post-breeding (orange), winter (light blue) and pre-breeding seasons (dark blue). General direction of post-breeding migration is indicated by the arrows (south via Davis Strait and Foxe Basin in orange) from 'post-breeding' to 'winter' and direction of pre-breeding migration is indicated by the arrow (north via Davis Strait in blue) from 'pre-breeding' to 'breed. The dashed line through the winter kernel represents a composite of the typical edge of the pack ice, 2010-2013, from December through April.

Twelve ivory gulls were captured using a modified version of a bownet trap [28] from a single colony on Seymour Island, Nunavut (Migratory Bird Sanctuary, Fig. 1; 78.80° N, 101.27° W) on 29 and 30 June, 2010. Five individuals were tagged with 20 g battery powered PTTs made by North Star Technologies (King Georges, Virginia). The remaining seven individuals were tagged with 15 g solar powered PTTs (a customized PTT-100 12 g model in a larger case to fit a larger solar chip) by Microwave Telemetry, Inc. (Columbia, Maryland). Individuals were caught during incubation to ensure they were actively breeding in Canada and a leg loop harness design was used to attach the transmitters, leaving flight muscles and major fat deposits unencumbered [29]. The transmitter plus the harness represented approximately 3% of ivory gull body mass; considered the maximum recommended load to minimize deleterious effects on individuals [30]. All birds successfully flew off after receiving the transmitters.

The PTTs were compatible with the Argos satellite positioning system [31]. The duty cycle of battery-powered PTTs was programmed to send signals within an 8 h period and shut off for 72 h. Solar powered PTTs had 10 hr on and 48 hr off with customized modifications to voltage by the manufacturer to accommodate the low incident light conditions of the Arctic fall and winter. Most data presented, notably data from March 2011 through July 2013, were from 4-5 solar-powered transmitters as all battery-powered units had failed by this time. Each message received from Argos was given an accuracy of the location estimate if four or more messages were sent to the satellite. We used only data with location class LC 1≤1500 m, LC 2≤500 m, or LC 3≤250 m. However, for generating kernel density maps and for analysing location in relation to ice cover, we include data with location class LC 0 (>1500 m). Kernels were produced using the reference bandwidth in the adehabitatHR package of Program R. If an activity sensor on the transmitter indicated that the bird was not moving (mortality or fallen off), we did not use any data from that transmitter collected after the date where the sensor indicated a problem. A description of how each PTT was powered, start and end dates of transmission and number of useable locations is in S1 Table.

We used data collected between 1 July 2010 and 30 June 2013. A blackout period occurred each year for approximately 8-10 weeks between November and January where the solar powered PTTs were not able to transmit data due to lack of sunlight at the latitude where the birds were wintering and consequently, insufficient recharging and power to send signals. Nonetheless, 19 720 locations were available for analyses of rates of travel; 59 439 locations were available for analyses of gull positions in relation to ice cover, and for generating density maps of gull locations by season.

### Data Processing

The statistical program R (version 2.15.1; R Foundation for Statistical Computing, Vienna, Austria) and ArcMap 10.1 (ArcMap; Environmental Systems Research Institute, Redlands, CA) were used for the analysis.

To properly assess how ivory gulls behaved during different times of the year, we estimated periods that described the breeding and wintering seasons as well as post-breeding and pre-breeding migrations. However, individual rates of travel and distances flown varied within and across years, and therefore defining a single date to begin and end a migration would introduce unnecessary bias. Arrival to the breeding and wintering areas were defined as the dates that a bird had obviously slowed down and was no longer making large directional movements (for the breeding season this was most often when they arrived in Parry Channel, above 74.5°N).

Defining the beginning of both post-breeding and pre-breeding migration for this species was subjective as ivory gulls have many short stopovers during their migration, and in some cases even traveled back in the direction from which they came. However, we defined the start of migration periods as the date when a bird began to fly long distances in an obvious linear pattern, generally away from the breeding or wintering area. Using the range of arrival and departure dates for individual ivory gulls, a median date was given to the beginning of each season (winter, pre-breeding migration, breeding and post-breeding migration) to standardize analyses across birds and years, as in Gilg et al. [32] shown in Table 1.

**Table 1:** Range of start dates, median start date and the median number of days and range for each season of the annual cycle for the 12 satellite-tagged ivory gulls throughout the study period, July 2010- July 2013.

Season	Median start date	Range	Median number of days (range)
Arrival to wintering area	19 Dec	20 Nov–17 Jan	154 (129–171)
Start of pre-breeding migration	15 May	02 May–28 May	18 (8–28)
Arrival to breeding area	05 Jun	23 May–19 Jun	118 (89–127)
Start of post-breeding migration	26 Sep	05 Sep–18 Oct	74 (50–121)

A second challenge included the blackout period of 24 h darkness between November and January when most tags transmitted few signals. Only two birds arrived at their wintering location before the blackout in late November (44517, 2010; 44523, 2012). One bird arrived at the wintering location in mid-January after the tags began transmitting again. The remaining birds had a data gap of one to three months, arriving at the wintering location at some point within the blackout period. Therefore the median date of arrival at the wintering location was taken to minimize bias.

Distance traveled was calculated as the orthodromic (great circle route) distance and was calculated in kilometres (km) between consecutive locations for each individual [32]. Rate of travel was then calculated from the distance and the time difference between two consecutive locations in km/h.

The final step before analysis of the data was to create a filter to extract any implausible rates of travel (∼flight speeds) for the birds. There were no reports of rates of travel for ivory gulls, only that they are reported to fly faster than black-legged kittiwakes (*Rissa tridactyla*) [14]. Oldén and Peterz [33] recorded black-legged kittiwake ground speeds of 96 km/h, so we used 100 km/h as the threshold rate of travel for ivory gulls. This was probably a liberal estimate, as birds never appeared to be flying this fast (M. Mallory, pers. observ.); however, a lower threshold may have removed satellite messages that were real bird movements, as wind-assisted flights could have resulted in true, high rates of travel. Wind data were not incorporated in this study.

### Data Analysis

Consecutive locations with time periods of less than 10 minutes were discarded as in Gilg et al. [19]. Mean hourly rates of travel (km/h) were then calculated for every month. Because most data distributions did not approximate normality (as assessed using Q-Q plots), we used non-parametric Kruskal-Wallis tests to assess whether the rates of travel of ivory gulls differed by season. A pairwise Wilcoxon test was then used to assess in which seasons the rates of travel varied significantly using the Holm [34] method to adjust the p-value for greater than three comparisons. All numbers were reported ± SD.

A weekly rate of travel index (km/wk) was also calculated using the median location per week for each ivory gull (Median Center tool in ArcGIS). This technique underestimated the distance flown over each period; however, using this approach standardizes the data to allow comparisons among individuals and seasons. As above, Kruskal-Wallis and pairwise Wilcoxon tests were run to compare weekly rates of travel among seasons.

To analyze sea ice concentration of dates ranging 01 July 2010 to 30 June 2013 in ArcGIS, the ‘Interpolate Time Series of Rasters at Points’ tool was used from the open source extension Marine Geospatial Ecology Tools [35]. This correlated daily sea ice charts (National Snow and Ice Data Center; http://nsidc.org/) with all ivory gull locations in relation to date. As the resolution of the sea ice charts were coarse (625 km^2^), the analysis included data points with accuracy of LC 0. Sea ice values of 254 and 253 indicated land, while values of 0 indicated no sea ice was present (i.e. open water) and all values in between represented sea ice concentration [36]. Three discreet habitat categories were used; ice (of any concentration), water and land. Proportions of time spent over each habitat type by each bird per year and season were calculated. To assess how proportions of birds over each habitat type changed across seasons, we used Kruskal-Wallis (K-W) tests followed by Dunn's Multiple Comparisons test when a significant KW analysis occurred. All numbers are reported ± SD.

## Results

### Geographical distribution

**Figure 2:**
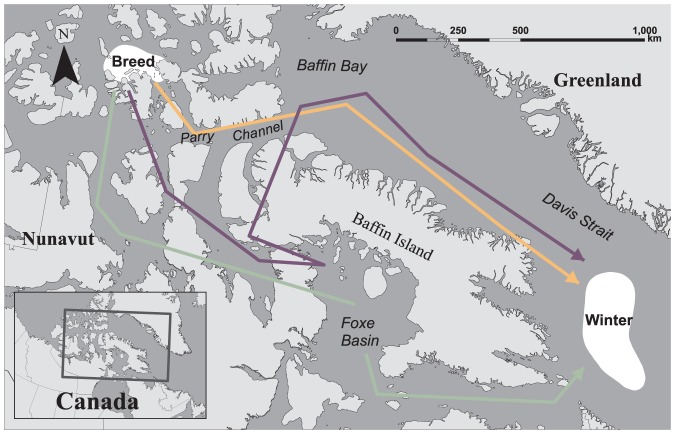
General direction of post-breeding migratory ivory gulls. a) Nine bird-years traveled south via Davis Strait (orange arrow); b) three bird-years traveled south via Foxe Basin Strait during and (green); and c) two bird-years traveled south via Foxe Basin, cutting north across Baffin Island and south through Davis Strait (purple) over the study period (July 2010- July 2013).

Ivory gulls breeding at the Seymour Island colony used areas north of Parry Channel to forage during late migration (pre-breeding, during breeding, and principally north and south of Parry Channel post-breeding (Fig. 1). Prior to migration, gulls moved into Parry Channel, Lancaster Sound (part of Parry Channel) and in one case up to the North Water Polynya. Foraging and migration locations varied among years, but key geographic regions that they used included Davis Strait, Foxe Basin, and (in one case) Hudson Bay (Fig. 2). Ivory gulls spent the winters in Davis Strait and the Labrador Sea. Ivory gulls rarely traveled over open water that was devoid of ice, but tended to remain near the pack ice (floe) edge in the winter. Only three transmissions were recorded within territorial waters (22 km) of Greenland, two being from the same individual (44526) on consecutive days in the spring and the other observation was during the fall (44523). The total area, defined by a maximum convex polygon, used by individuals during the study was 6 005 588 km^2^.

### Sea ice

**Figure 3:**
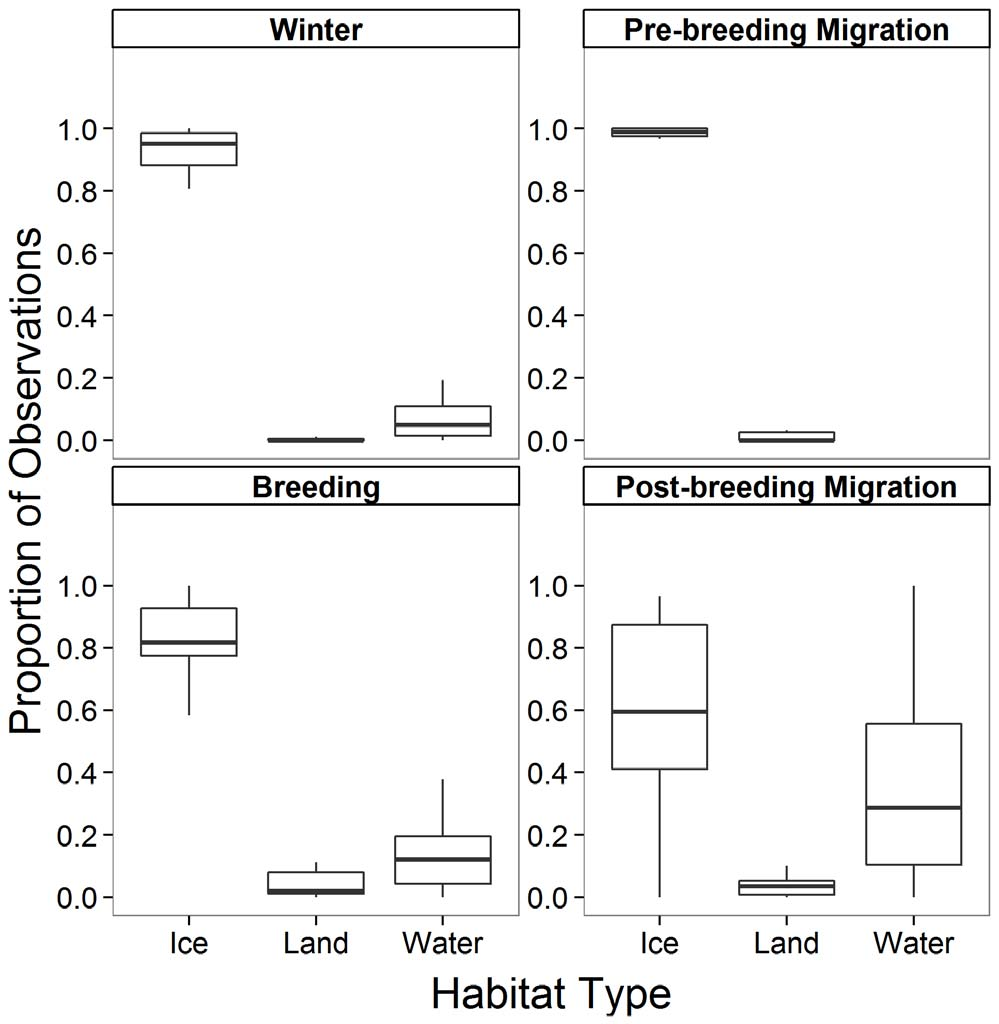
Median and 25^th^ and 75^th^ quartiles of the proportion of observations of the 12 satellite-tagged ivory gulls over ice, land or water in winter (n = 15 bird-seasons, 403 days), b) pre-breeding migration (n = 12, 62), c) breeding (n = 21, 337) and d) post-breeding migration (n = 19, 140). Whiskers represent values within 1.5 times the interquartile range. Total number of days with observations was 942 over the study period, July 2010- July 2013.

Across all years and individuals (n = 12 birds), ivory gulls had three habitat types over which they could be recorded: sea ice, land, or open water. Time spent over ice, land and water differed among seasons (K-W test, χ^2^ = 24.4, p<0.0001; χ^2^ = 14.2 p = 0.003; χ^2^ = 28.6, p<0.0001, respectively; Fig. 3). Ivory gulls spent less time over sea ice in the breeding season than during pre-breeding migration (Dunn's Multiple Comparisons test, p<0.01), whereas post-breeding migration showed that fewer individuals spent time over ice compared with pre-breeding migration (p<0.001) and winter (p<0.01). Time spent over land in winter was significantly less than during breeding (p<0.05) and post-breeding migration (p<0.01). Finally, time spent over water during the breeding season was greater than during pre-breeding migration (p<0.001) and more time was spent over water compared to winter (p<0.05) and pre-breeding migration (p<0.001).

### Annual means of ivory gull movements

Using data from four ivory gulls that provided at least one full year of information (all solar powered transmitters), the average ivory gull had a mean hourly rate of travel of 6.2±8.9 km/h (n = 10 bird-years) and an average weekly movement of 185±258 km/wk. The maximum annual distance flown was 19 600–46 600 km; these estimates included some points rated as accuracy LC 0, but also had several weeks of no data transmissions due to the blackout period in November/December, so the actual annual distance flown was probably within this range. Individually, however, gull behaviour varied among season, individual and year.

### Seasonal spatial and temporal patterns

#### Wintering

Ivory gulls stayed in Davis Strait and the Labrador Sea for a median duration of 154 d (129–171 d) during the winter. Median date of arrival was 19 December (range 20 November – 17 January). One ivory gull (44509) remained in Barrow Strait (part of Parry Channel; 76.05°N -105.01°W) during the winter of 2010, until the transmitter failed on 23 January 2011. The activity sensor indicated that this bird remained alive until that day.

#### Pre-breeding migration

Pre-breeding migration occurred over 18 d (8–28 d). The earliest date of departure was 2 May and the latest departure was 28 May. Gulls flew relatively directly to breeding colonies and with few stops during migration. Throughout all years tracked during pre-breeding migration (six birds, n = 12 bird-years), ivory gulls only travelled north though Davis Strait and Baffin Bay, then flew west though Lancaster Sound and subsequently passed Cornwallis Island to get to their breeding area.

#### Breeding

**Figure 4:**
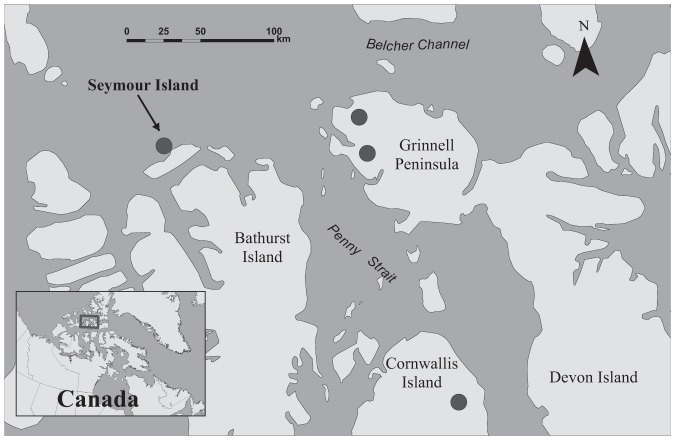
Breeding colonies used in 2011 and 2013 by ivory gulls. In 2011, one was located on Seymour Island, at the long-known colony site (both gulls abandoned in incubation) and two newly found colonies were located on Grinnell Peninsula (one ivory gull at each colony). In 2013, one ivory gull was located on Cornwallis Island, but transmissions ceased shortly after its arrival.

All ivory gulls abandoned breeding after being tagged in 2010. After pre-breeding migration, birds foraged in the vicinity of the colony for a median of 10 d (7–26 d). Two individuals appeared to have bred successfully in 2011 (44523 and 44525), as indicated by the amount of time spent at the breeding colony (52 d and 65 d, respectively). Both individuals bred at separate colonies that had not been previously discovered, located on Grinnell Peninsula of Devon Island (Fig. 4). However, 44523 visited Seymour Island between 23–27 June. Gulls 44524 and 44530 both arrived on Seymour Island in 2011 and appeared to initiate breeding but subsequently abandoned. Of four gulls still transmitting, none bred in 2012, but 44525 landed at a known colony on eastern Devon Island before the satellite tag stopped transmitting on the day it arrived at the colony (the activity sensor also began indicating that there was no movement later in the day that it arrived). This suggests that the bird died after arriving at this site. Gull 44526 stopped on eastern Cornwallis in 2013 at a previously unknown breeding site, just north of some known colonies; however, the activity sensor indicated that the transmitter was no longer moving one day after arriving at the colony, meaning the gull had either died or the transmitter had been detached (Fig. 4).

#### Non-breeding

Ivory gulls that did not breed foraged as far north as 82° N, extending north to Ellesmere Island, west to Victoria Island and Parry Channel until post-breeding migration. The area used by non-breeding ivory gulls originally tagged at Seymour Island was 1 239 571 km^2^, while breeding satellite-tagged ivory gulls used a smaller area of 38 541 km^2^ for the study period July 2010 to July 2013. Non-breeding birds remained in the breeding area between 23 May and 17 October (median 79 d, range 12–127 d).

#### Post-breeding migration

Ivory gulls left the breeding area at varying times between 5 September and 18 October (median date 26 September). The median length of the post-breeding migration was 74 d (range 50–121 d). Migratory movements were punctuated by regular stops, presumably to forage, rather than long, continuous flights observed in pre-breeding migration. Ivory gulls flew one of three routes during post-breeding migration: 1) east though Lancaster Sound and then south though Baffin Bay and Davis Strait (orange arrow, Fig. 2); 2) south from Lancaster Sound though Prince Regent Inlet and Foxe Basin (green arrow, Fig. 2); and 3) a combination of moving south through Prince Regent Inlet, north to Baffin Bay and south through Davis Strait (purple arrow, Fig. 2). Individuals exhibited variation in post-breeding migration: one gull used the Baffin Bay/Davis Strait route three years in a row, while three other gulls used different routes in successive years (S2 Table).

#### Rate of Travel

**Figure 5:**
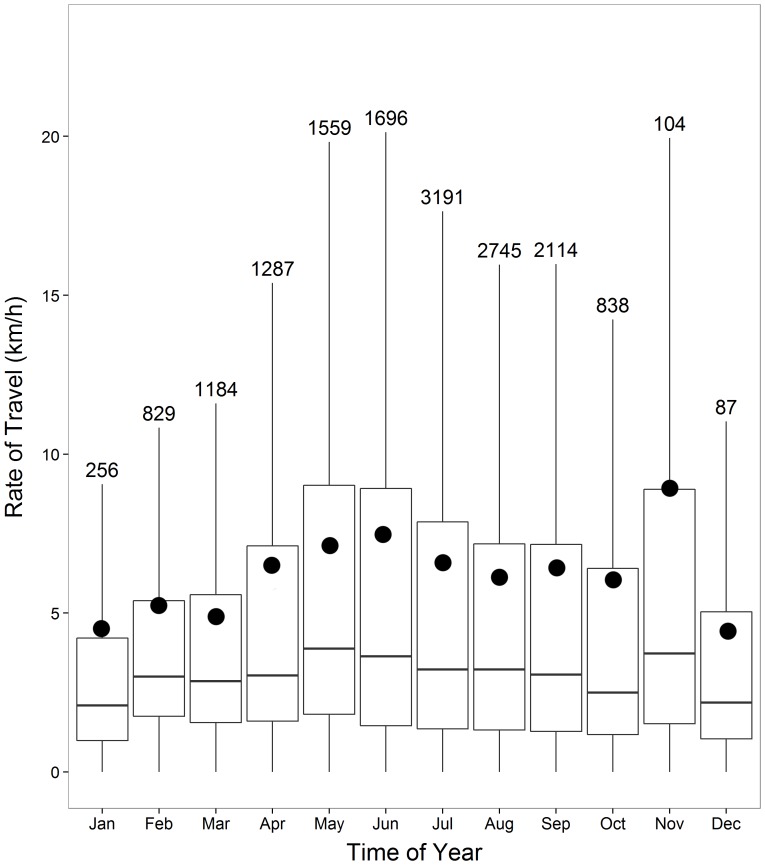
Hourly rates of travel (km/hr) per month. Box plots show median and 25^th^ and 75^th^ quartiles for all satellite-tagged ivory gulls throughout the study period, July 2010- July 2013. Whiskers represent values within 1.5 times the interquartile range. Mean values are depicted by the black circles. Note small sample size for November and December due to too few records during the blackout period for the transmitters.

While mean hourly rate of travel for ivory gulls across all months was 6.2±8.9 km/h (range 0–99 km/h), peak hourly rates of travel appeared during November (n = 104 bird-days; 9.2±14.5 km/h) when all birds had initiated migration (Fig. 5). Between January (n = 256) and April (n = 1287), ivory gulls flew at average speeds between 4.6±8.8 km/h to 6.5±10.1 km/h, which increased through June (n = 1696; 7.7±11.3 km/h), and then decreased slightly through the breeding and post-breeding season (Fig. 5). Collectively, ivory gulls flew at significantly different hourly rates of travel across seasons (K-W test, χ^2^_11_ = 61.1, p<0.0001). Considering only the “breeding season” period (∼summer), mean rates of travel of non-breeding individuals were significantly lower (n = 5095; 6.0±8.5 km/h) from those birds that were breeding (n = 4211; 6.9±9.9 km/h; p = 0.018).

**Figure 6:**
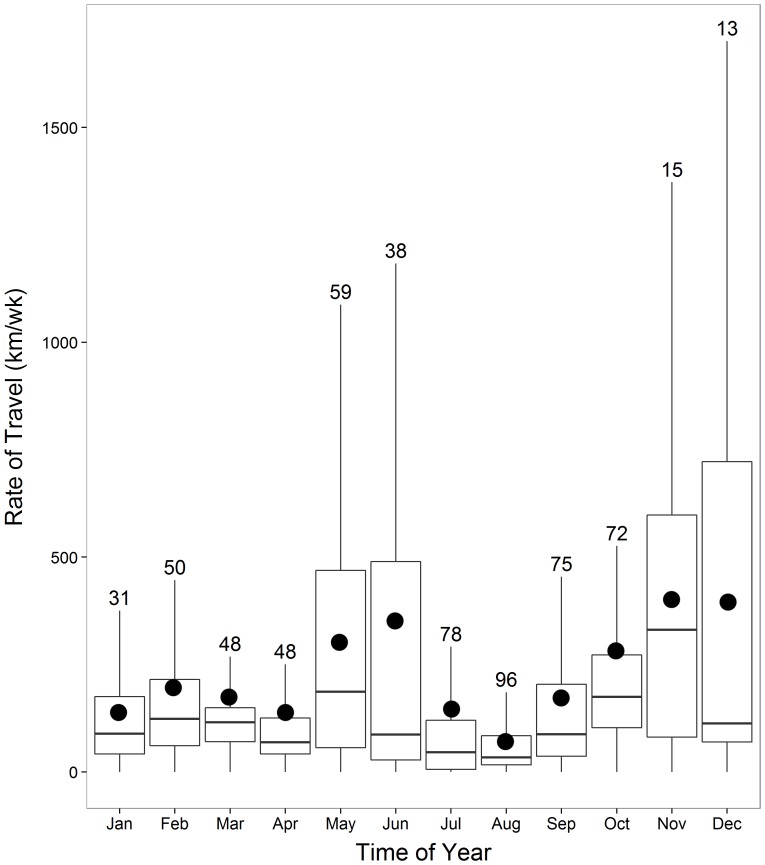
Weekly rates of travel (km/wk) per month. Box plots show median and 25^th^ and 75^th^ quartiles for all satellite-tagged ivory gulls throughout the study period, July 2010- July 2013. Whiskers represent values within 1.5 times the interquartile range. Mean values are depicted by the black circles. Note small sample sizes for November and December due to too few records during the blackout period for the transmitters.

Mean indices of weekly rates of travel differed significantly through the year (K-W test, χ^2^_3_ = 89.0, p<0.0001; Fig. 6) as well, with weekly travel during each season (breeding: n = 268; 212±381 km/wk; post-breeding: n = 109; 255±255 km/wk; winter: n = 211; 163±209 km/wk; pre-breeding: n = 35; 393±325 km/wk) being significantly different from each other season (Pairwise Wilcoxon; all p<0.001).

## Discussion

Satellite tracking of ivory gulls provided novel information to their movement patterns and annual habitat needs. As expected, the breeding distribution was consistent with previous banding and observational work [12], [15]. However, their wintering distribution has been speculative, based on intermittent observations and a single winter survey [10]. Our results confirm that Davis Strait and the Labrador Sea form the main annual winter areas for ivory gulls from Seymour Island, supporting earlier survey work [10], [11], [19] which showed that ivory gulls from Russia, Greenland and Svalbard also use the northwest Atlantic to winter. Collectively, these findings suggest that this area is of international significance to the ivory gull. Given the proximity of the main wintering region to the recurrent pack ice edge (Fig. 1), we believe that Davis Strait and the Labrador Sea are important for wintering ivory gulls because they provide a predictable food supply along the pack ice edge. Here, gulls may scavenge polar bear kills, forage on leftovers from hooded seal whelping patches, or prey on marine, ice-associated fish (e.g. Arctic cod *Boreogadus saida*) and sympagic invertebrates along the ice edge and leads [10], [13], [14].

### Migration and sea ice

Our data showing the distribution of ivory gulls during spring and fall have markedly changed our perception of ivory gull migration, including their heavy reliance on sea ice formation and recession. It has long been believed that Canadian ivory gulls migrate principally along the Greenland coast [27]. However, our study found that individuals showed great plasticity in their migration routes and timing, which can likely be attributed to their affinity for sea ice and their apparent avoidance of open water [14], [16], [19]. It is possible that our recent tracking work has documented a shift in the migratory movements of ivory gulls from Seymour Island compared to prior banding results, as a response to changing ice conditions or to years of human harvest in Greenland [5], [27]. Sea ice formation begins around October (when the majority of ivory gulls initiated fall migration) and extends slowly south through Davis Strait until it has reached its full extent in March [36]. The formation of sea ice in the fall may be a contributing factor to the variety of migratory routes that ivory gulls take, selecting those that offer the best foraging opportunities. For example, Gehrold et al. [37] showed that migrating European gadwalls (*Ana strepera*) exhibit individual variation in migratory movements, exploiting productive feeding grounds. Second, sea ice extent may explain, in part, why fall migration was four times longer than spring migration, as individuals made many stopovers in the fall (likely to forage), moving quickly between the stops and rarely extending east of the ice edge. In contrast, pre-breeding migration was comparatively short and weekly rates of travel sometimes high. Ivory gulls moved west of the receding sea ice in May of each year of the study, back to the breeding area where ice was still heavily concentrated [36]. The timing of sea ice recession clearly influences spring migration in other Arctic seabirds (e.g. [38]) and the specific association with sea ice formation and recession during migratory periods has also been observed in another Arctic species, the Bewick's swan (*Cygnus columbianus bewickii*), and is likely correlated with food availability [39].

How are ivory gulls able to survive or even exploit extensive ice cover during migration? First, the combination of wind and water currents keep polynyas and shore leads of varying sizes open during migration periods and the winter [40]. Thus, gulls likely seek out these sites as they move, because they provide open water access to marine fish and invertebrate prey [1], [11], [13], [40], and possibly because gulls may exploit scavenging opportunities from polar bears hunting near these sites [41]. Secondly, primary productivity and associated foraging “hotspots” are intricately tied to the links between landfast ice, ice edges, epontic algae growth, snow cover on the ice, and solar irradiance [40], [42], [43]. These factors collectively lead to patches of prey richness and foraging opportunities in an apparent endless sea of ice, and ivory gulls may also exploit these locations (like many other Arctic marine birds) [44] to prey on marine fish and invertebrates [13], [41], [42] or scavenge from bear kills of abundant seal populations [45]. In fact, Inuit local ecological knowledge showed that ivory gulls nesting on northwestern Baffin Island stopped annually during late migration to scavenge on Inuit-killed marine mammal carcasses left on the sea ice near Arctic Bay, Nunavut [46]. Alternatively, many Arctic nesting migrants use residual body stores from the wintering sites or stopovers to survive until the ice has broken up [47] and although this may not be their sole strategy, it may have an effect on their reproductive success [48].

### Breeding and non-breeding seabirds

For a long-lived seabird, exhibiting breeding site fidelity may depend on age, costs associated with changing nest sites, reproductive success in the previous year, probability of adult mortality, and the individual's knowledge of other colonies [49]–[51]. Despite the lack of reproductive success in 2010 and the disturbance caused by tagging the ivory gulls, two individuals returned to the Seymour Island colony in 2011. Conover and Miller [51] suggested that when there are few suitable habitats located in the vicinity for nesting, ring-billed gulls (*Larus delawarensis*) should remain at their current nesting site, regardless of previous predation history. Neighbours at the Seymour Island colony in 2010 may have been successful; a cue to the disturbed individuals to return the following year [49]. However, in 2011, we suspect that Seymour Island was probably disturbed by mammalian predators (which occurs frequently; [14]). One individual abandoned after 11 days spent at the colony and the other did not continue transmitting. The two individuals that switched nest sites in 2011 likely chose higher quality habitats for breeding, located to the west on Grinnell Peninsula of northwest Devon Island; previously undiscovered nesting sites. A 2011 survey found these colonies supported 69 birds, representing >8% of the estimated Canadian population in an area previously unknown as an ivory gull nesting location [26]. This suggests that current population numbers may be underestimates and that there may be other undiscovered colonies elsewhere. Ivory gulls, like ring-billed gulls, likely arrive at the breeding area in the spring to predict the current situation at the breeding colony and may use previous outcomes as well as current environmental conditions to dictate whether they will breed or not [52].

### Rate of Travel

Our study found that ivory gulls traveled an average of 6.3 km/hr at any time of the year. This number is slightly lower than hourly rates calculated for northeast Atlantic populations of the ivory gull (∼10 km/hr) [19], using similar analytical methods to generate a valid comparison. Furthermore, ivory gulls from the northeast Atlantic exhibited their highest rates of travel in November ([19]; although that study only considered the second half of a calendar year), and in our study, the greatest rates of travel were also for birds migrating in November. In fact, the seasonal patterns of hourly and weekly rates of travel for Canadian ivory gulls were very similar to that found by Gilg et al. [19]. Thus, these two studies from different breeding centres suggest similar movement behaviours of the gulls. However, Gilg et al. [19] reported an average distance traveled for July to December for Greenland ivory gulls of 7 000–50 000 km based on the ivory gull rates of travel from that period. This is a very broad range and a different estimate than the maximum annual travel we calculated for one Canadian ivory gull (46 600 km), and likely differs because the calculations were based on extrapolation from individual rates of travel during a smaller time period.

The study period of Gilg et al. [19] covered July to December and therefore we could not compare rates of travel at the overwintering sites. However, ivory gulls in our study moved more than expected, averaging 163 km/wk. Although there are few studies that have examined winter energetics, the little auk (*Alle alle*) and Brunnich's guillemot (*Uria lomvia*) have a large increase in energetic requirements after December, remaining high throughout the winter in the northwest Atlantic [53]. Similar to these seabirds, ivory gulls likely require more energy to compensate for the environmental challenges (i.e. storms, increased thermoregulatory needs) occurring in winter [53], and thus, ivory gulls appeared to be searching almost constantly for foraging opportunities.

### Comparisons among other seabirds

The ivory gull is rare in the seabird world being the only species, other than the black guillemot *(Cepphus grylle*), capable of remaining north of 70°N in the winter [54]. In comparison with other Canadian high Arctic-nesting seabirds, they migrate short distances and do not appear to have migratory staging areas, unless they were used during the blackout periods of the study [55], [56] (northeast Atlantic ivory gulls do use staging areas during fall migration, [19]). In contrast, Arctic breeding gulls that are long distance migrants, such as the black-legged kittiwake (*Rissa tridactyla*) and Sabine's gull (*Xema sabini*), use staging areas pre- and post-breeding [57], [58]. Long distance migrants should be less selective for favourable weather, due to the costs of slowing migration progress and rates of travel and may explain why the black-legged kittiwake and Sabine's gull travel approximately 50 km/hr greater than the ivory gull during migration [47], [59]. As well, staging areas for molting may be unnecessary for Canadian ivory gulls as the waters remain open in the breeding area until October, allowing continued foraging opportunities as molting resumes [60]. Gilg et al. [19] noted that some ivory gulls of northeast Atlantic populations also remained close to their breeding colony until the end of September. The availability of prey near the breeding colony well into the fall may explain the varied dates of departure of the Canadian ivory gull.

The ivory gull's relationship with sea ice is apparent throughout the post-breeding migratory period, likely altering their movements according to sea ice formation. This close association with dense pack ice (on average 50% concentration) and ice edges was clear in all seasons and years for all individuals; a well-documented relationship that is not known in other seabirds [10], [11], [23], except its analogue in the Antarctic, the snow petrel (*Pagodroma nivea*) [61]. Ivory gulls rarely flew over land, except one instance where a gull flew almost 500 km within 6 h across Baffin Island to access the next ice-covered destination. This affinity for moving over ice may be part of a strategy to avoid land-based predators (similar to their breeding strategy) [14], or to maximize their chances of having access to sympagic prey items and scavenging bear kills [11], [13].

Arctic seabirds, like the ivory gull, live in a dynamic and harsh environment that is experiencing dramatic changes due to global warming. The overall warming trend in ocean temperatures will affect the spatial and temporal distribution of seabird prey [62], [63], factors known to influence reproductive phenology and success in some other Arctic seabird species (e.g., Mallory et al. [8]). Like Gilg et al. [19] for northeast Atlantic birds, our study has established a baseline for timing of movements and habitat preferences of ivory gulls in their annual cycle in Canada, and there is a suggestion that fall migration routes may differ from ones of birds banded in the 1980s [27]. However, it remains unclear whether the gulls have the behavioral plasticity to deal with predicted declines in the extent and duration of sea ice in the coming years, and if they do, what consequences this may have for body condition and reproduction. Like the predictions for polar bears [64], [65], recent declines in ivory gull populations [3], [15], [26] may be a harbinger of the future for ice-associated species in the Arctic.

## Supporting Information

S1 Table Description of each satellite transmitter (PTT number) and how the device was powered (battery or solar). The dates the transmitter ran is included as well as the total number of months for which data were collected and a count of the good quality location records (LC 1, 2 and 3) that were available for analysis for the study period, July 2010- July 2013.
(DOCX)

S2 Table Individual dates of arrival/departure for 12 satellite-tagged ivory gulls as well as migratory routes and breeding colonies used throughout respective annual cycles throughout the study period, July 2010- July 2013. ‘No Attempt’ indicates that no breeding attempt was made during the breeding season.
(XLS)
