# Supplementary material for: Annual Movement Patterns of Endangered Ivory Gulls: The Importance of Sea Ice
Source: PLoS One. 2014 Dec 31;9(12):e115231. doi: 10.1371/journal.pone.0115231 (PMC4281149; doi:10.1371/journal.pone.0115231)
Supplement: S1 Table — Description of each satellite transmitter (PTT number) and how the device was powered (battery or solar). The dates the transmitter ran is included as well as the total number of months for which data were collected and a count of the good quality location records (LC 1, 2 and 3) that were available for analysis for the study period, July 2010- July 2013. (DOCX) [file pone.0115231.s001.docx]

**Table S1**: Description of each satellite transmitter (PTT number) and how the device was powered (battery or solar). The dates the transmitter ran is included as well as the total number of months for which data were collected and a count of the good quality location records (LC 1, 2 and 3) that were available for analysis for the study period, July 2010- July 2013.

| **PTT** | **Battery/ solar power** | **Dates running** | **# months transmitting** | **Useable locations (LC 1,2,3)** |
| --- | --- | --- | --- | --- |
| 44509 | Battery | 1 Jul, 2010 – 23, Jan, 2011 | 7 | 360 |
| 44516 | Battery | 1 Jul, 2010 – 18, Nov, 2010 | 5 | 263 |
| 44517 | Battery | 1 Jul, 2010 – 18, Feb, 2011 | 8 | 400 |
| 44519 | Battery | 1 Jul, 2010 – 03, Mar, 2011 | 8 | 394 |
| 44522 | Battery | 1 Jul, 2010 – 26, Nov, 2010 | 5 | 304 |
| 44523 | Solar | 1 Jul, 2010 - current date | 48 + | 5677 |
| 44524 | Solar | 1 Jul, 2010 – 26, Sep, 2012 | 38 | 4307 |
| 44525 | Solar | 1 Jul, 2010 – 20, Aug, 2012 | 38 | 3461 |
| 44526 | Solar | 1 Jul, 2010 – 30, May, 2013 | 49 | 1694 |
| 44529 | Solar | 1 Jul, 2010 – 22, Nov, 2010 | 5 | 141 |
| 44530 | Solar | 1 Jul, 2010 – 11, Jul, 2011 | 12 | 1406 |
| 44531 | Solar | 1 Jul, 2010 – 20, May, 2011 | 11 | 1313 |
